# Supplementary material for: Culture matters: A systematic review of antioxidant potential of tree legumes in the semiarid region of Brazil and local processing techniques as a driver of bioaccessibility
Source: PLoS One. 2022 Mar 9;17(3):e0264950. doi: 10.1371/journal.pone.0264950 (PMC8906597; doi:10.1371/journal.pone.0264950)
Supplement: S2 Table — (DOCX) [file pone.0264950.s002.docx]

**Supporting Information, File 1**

**Studies on Ethnobiology**

1. Andrade-Lima, D., 1954. Contribution to the Study of the Flora of Pernambuco, Brazil. State University of New York.
2. Cartaxo, S.L., de Almeida Souza, M.M., de Albuquerque, U.P., 2010. Medicinal plants with bioprospecting potential used in semi-arid northeastern Brazil. J. Ethnopharmacol. 131, 326–342. https://doi.org/10.1016/j.jep.2010.07.003
3. de Sousa Araújo, T.A., Alencar, N.L., de Amorim, E.L.C., de Albuquerque, U.P., 2008. A new approach to study medicinal plants with tannins and flavonoids contents from the local knowledge. J. Ethnopharmacol. 120, 72–80. https://doi.org/10.1016/j.jep.2008.07.032
4. Leal, J.B., Silva, M.M. da, Costa, J.M., Albuquerque, L.C. da S. de, Pereira, M. das G. da S., Sousa, R.L. de, 2019. Etnobotânica de plantas medicinais com potencial anti- inflamatório utilizadas pelos moradores de duas comunidades no município de Abaetetuba, Pará. Biodiversidade 3, 110–125.
5. Macedo, J.G.F., De Menezes, I.R.A., Ribeiro, D.A., Santos, M.D.O., De Mâcedo, D.G., Macêdo, M.J.F., De Almeida, B.V., Souza De Oliveira, L.G., Pereira Leite, C., De Almeida Souza, M.M., 2018. Analysis of the Variability of Therapeutic Indications of Medicinal Species in the Northeast of Brazil: Comparative Study. Evidence-based Complement. Altern. Med. 2018. https://doi.org/10.1155/2018/6769193
6. Macêdo, M.J.F., Ribeiro, D.A., Santos, M. de O., Macêdo, D.G. de, Macedo, J.G.F., Almeida, B.V. de, Saraiva, M.E., Lacerda, M.N.S. de, Souza, M.M. de A., 2018. Fabaceae medicinal flora with therapeutic potential in Savanna areas in the Chapada do Araripe, Northeastern Brazil. Rev. Bras. Farmacogn. 28, 738–750. https://doi.org/10.1016/j.bjp.2018.06.010
7. Magno-Silva, E.R., Rocha, T.T., Tavares-Martins, A.C.C., 2020. Ethnobotany and ethnopharmacology of medicinal plants used in communities of the soure marine extractive reserve, Pará State, Brazil. Bol. Latinoam. y del Caribe Plantas Med. y Aromat. 19, 29–64.
8. Mesquita, U.D.O., Tavares-Martins, A.C.C., 2018. Etnobotánica de plantas medicinales en la comunidad de Caruarú, Isla del Mosqueiro, Belém-PA, Brasil. Bol. Latinoam. y del Caribe Plantas Med. y Aromat. 17, 130–159.
9. Nascimento, V., Vasconcelos, M., Maciel, M., Albuquerque, U., 2012. Famine Foods of Brazil’s Seasonal Dry Forests: Ethnobotanical and Nutritional Aspects. Econ. Bot. 66, 22–34. https://doi.org/10.1007/s12231-012-9187-2
10. Nunes, E.N., Guerra, N.M., Arevalo-Marin, E., Alves, C.A.B.A.B., do Nascimento, V.T., da Cruz, D.D., Ladio, A.H., Silva, S. de M., de Oliveira, R.S., de Lucena, R.F.P.P., Arévalo-Marín, E., Alves, C.A.B.A.B., do Nascimento, V.T., da Cruz, D.D., Ladio, A.H., Silva, S. de M., de Oliveira, R.S., de Lucena, R.F.P.P., 2018. Local botanical knowledge of native food plants in the semiarid region of Brazil. J. Ethnobiol. Ethnomed. 14. https://doi.org/10.1186/s13002-018-0249-0
11. Palheta, I.C., Tavares-Martins, A.C.C., Lucas, F.C.A., Jardim, M.A.G., 2017. Ethnobotanical study of medicinal plants in urban home gardens in the city of abaetetuba, Pará state, Brazil. Bol. Latinoam. y del Caribe Plantas Med. y Aromat. 16, 206–262.
12. Ribeiro, D.A., Oliveira, L.G.S. De, Macêdo, D.G. De, Menezes, I.R.A. De, Costa, J.G.M. Da, Silva, M.A.P. Da, Lacerda, S.R., Souza, M.M.D.A., 2014. Promising medicinal plants for bioprospection in a Cerrado area of Chapada do Araripe, Northeastern Brazil. J. Ethnopharmacol. 155, 1522–1533. https://doi.org/10.1016/j.jep.2014.07.042
13. Ribeiro, R.V., Bieski, I.G.C., Balogun, S.O., Martins, D.T. de O., 2017. Ethnobotanical study of medicinal plants used by Ribeirinhos in the North Araguaia microregion, Mato Grosso, Brazil. J. Ethnopharmacol. 205, 69–102. https://doi.org/10.1016/j.jep.2017.04.023
14. Santos, E.Q., Costa, J.F.D.S., Pereira, M. das G.D.S., Costa, J.M., De Sousa, R.L., 2019. Etnobotânica da flora medicinal de quintais na comunidade Mamangal, Rio Meruú, Igarapé-Miri, Pará. Sci. Plena 15. https://doi.org/10.14808/sci.plena.2019.051202
15. Saraiva, M.E., Ulisses, A.V.R.D.A., Ribeiro, D.A., Oliveira, L.G.S. De, Macêdo, D.G. De, Sousa, F.D.F.S. De, Menezes, I.R.A. De, Sampaio, E.V.D.S.B., Souza, M.M.D.A., 2015. Plant species as a therapeutic resource in areas of the savanna in the state of Pernambuco, Northeast Brazil. J. Ethnopharmacol. 171, 141–153. https://doi.org/10.1016/j.jep.2015.05.034
16. Souza, L.F., Dias, R.F., Guilherme, F.A.G., Coelho, C.P., 2016. Plantas medicinais referenciadas por raizeiros no município de Jataí, estado de Goiás. Rev. Bras. Plantas Med. 18, 451–461. https://doi.org/10.1590/1983-084X/15_173
17. Souza, R.K.D., Silva, M.A.P. Da, Menezes, I.R.A. De, Ribeiro, D.A., Bezerra, L.R., Souza, M.M.D.A., 2014. Ethnopharmacology of medicinal plants of Carrasco, northeastern Brazil. J. Ethnopharmacol. 157, 99–104. https://doi.org/10.1016/j.jep.2014.09.001

**Studies on Chemical Profile**

1. Aguiar, J.C.D., Santiago, G.M.P., Lavor, P.L., Veras, H.N.H., Ferreira, Y.S., Lima, M.A.A., Arriaga, Â.M.C., Lemos, T.L.G., Lima, J.Q., Jesus, H.C.R. de, Alves, P.B., Braz-Filho, R., 2010. Chemical Constituents and Larvicidal Activity of Hymenaea courbaril Fruit Peel. Nat. Prod. Commun. 1, 9–12.
2. Andrade, B. de A., Corrêa, A.J.C., Gomes, A.K.S., Neri, P.M. da S., Sobrinho, T.J. da S.P., Araújo, T.A. de S., Castro, V.T.N. de A. e, Amorim, E.L.C. de, 2019. Photoprotective activity of medicinal plants from the caatinga used as anti-inflammatories. Pharmacogn. Mag. 15, 356–361.
3. Araújo, A.A. de, Soares, L.A.L., Assunção Ferreira, M.R., De Souza Neto, M.A., Da Silva, G.R., De Araújo, R.F., Guerra, G.C.B., De Melo, M.C.N., 2014. Quantification of polyphenols and evaluation of antimicrobial, analgesic and anti-inflammatory activities of aqueous and acetone-water extracts of Libidibia ferrea, Parapiptadenia rigida and Psidium guajava. J. Ethnopharmacol. 156, 88–96. https://doi.org/10.1016/j.jep.2014.07.031
4. Azevedo, L.F.C. de, Ferreira, T.A.A., Melo, K.M., Dias, C.L.P., Bastos, C.E.M.C., Santos, S.F., Alberdan da Silva Santos, Nagamachi, C.Y., Pieczarka, J.C., 2020. Aqueous ethanol extract of Libidibia ferrea (Mart. Ex Tul) L.P. Queiroz (juca) exhibits antioxidant and migration-inhibiting activity in human gastric adenocarcinoma (ACP02) cells. PLoS One 15, e0226979.
5. Barros, A.O., De Souza, R.S., Aranha, E.S.P., Da Costa, L.M., De Souza, T.P., De Vasconcellos, M.C., Lima, E.S., 2014. Antioxidant and hepatoprotective activities of libidibia ferrea bark and fruit extracts. Int. J. Pharm. Pharm. Sci. 6, 71–76.
6. Bezerra, G.P., Góis, R.W.D.S., Brito, T.S. De, Lima, F.J.B. De, Bandeira, M.A.M., Romero, N.R., Magalhães, P.J.C., Santiago, G.M.P., 2013. Phytochemical study guided by the myorelaxant activity of the crude extract, fractions and constituent from stem bark of Hymenaea courbaril L. J. Ethnopharmacol. 149, 62–69. https://doi.org/10.1016/j.jep.2013.05.052
7. Bhattacharyya, J., Batista, J.S., Almeida, R.N., 1995. Dioclein, a flavanone from the roots of Dioclea grandiflora. Phytochemistry 38, 277–278. https://doi.org/10.1016/0031-9422(94)00528-2
8. Bhattacharyya, J., Majetich, G., Jenkins, T.M., Almeida, R.N., 1998. Dioflorin, a minor flavonoid from Dioclea grandiflora. J. Nat. Prod. 61, 413–414. https://doi.org/10.1021/np9704314
9. Bhattacharyya, J., Majetich, G., Spearing, P., Almeida, R.N., 1997. Dioclenol, a minor flavanonol from the root-bark of Dioclea grandiflora. Phytochemistry 46, 385–387. https://doi.org/10.1016/S0031-9422(97)00297-5
10. Comandolli-Wyrepkowski, C.D., Jensen, B.B., Grafova, I., dos Santos, P.A., Comapa Barros, A.M., Soares, F.V., Marques Barcellos, J.F., da Silva, A.F., Grafov, A., Ramos Franco, A.M., 2017. Antileishmanial activity of extracts from Libidibia ferrea: development of in vitro and in vivo tests. ACTA Amaz. 47, 331–340. https://doi.org/10.1590/1809-4392201700871
11. Falcão, T.R., Araújo, A.A. de, Soares, L.A.L., Farias, I.B. de, Silva, W.A.V. da, Ferreira, M.R.A., Jr, R.F. de A., Medeiros, J.S. de, Lopes, M.L.D. de S., Guerra, G.C.B., 2019a. Libidibia ferrea fruit crude extract and fractions show anti-inflammatory, antioxidant, and antinociceptive effect in vivo and increase cell viability in vitro. Evidence-based Complement. Altern. Med. https://doi.org/https://doi.org/10.1155/2019/6064805
12. Falcão, T.R., Rodrigues, C.A.O., De Araújo, A.A., de Medeiros, C.A.C.X., Soares, L.A.L., Ferreira, M.R.A., Vasconcelos, R.C., De Araújo Júnior, R.F., De Sousa Lopes, M.L.D., Guerra, G.C.B., Falcao, T.R., Oliveira Rodrigues, C.A., de Araujo, A.A., de Medeiros, C.A.C.X., Lira Soares, L.A., Assuncao Ferreira, M.R., Carvalho Vasconcelos, R., de Araujo Junior, R.F., De Sousa Lopes, M.L.D., Bernardo Guerra, G.C., 2019b. Crude extract from Libidibia ferrea (Mart. ex. Tul.) LP Queiroz leaves decreased intra articular inflammation induced by zymosan in rats. BMC Complement. Med. Ther. 19. https://doi.org/10.1186/s12906-019-2454-3
13. Ferreira, D.Q., Ferraz, T.O., Araújo, R.S., Alves, R., Cruz, S., Fernandes, C.P., Souza, G.C., Ortiz, B.L.S., Sarquis, R.S.F.R., Miranda, J.C.M.M., Garrett, R., Tavares Carvalho, J.C., Eliza, A., De Faria, M., Oliveira, M., 2019. Libidibia ferrea (jucá), a Traditional Anti-Inflammatory: A Study of Acute Toxicity in Adult and Embryos Zebrafish (Danio rerio). mdpi.com. https://doi.org/10.3390/ph12040175
14. Ferreira, M.R.A., Fernandes, M.T.M., Wliana A. V. da Silva, Bezerra, I.C.F., Tatiane P. de Souza, Pimentel, M.F., Soares, L.A.L., 2016. Chromatographic and Spectrophotometric Analysis of Phenolic Compounds from Fruits of Libidibia ferrea Martius. Pharmacogn. Mag. 12, S285-91.
15. Ferreira, M.R.A., Sousa, P.A., Machado, J.C.B., Soares, L.A.L., 2020. Extraction of monomers of hydrolysable tannins from PODS of libidibia ferrea (Mart. ex Tul.) L. P. Queiroz: Effects of solvent and amount of drug using response-surface methodology and desirability profile. Quim. Nova 43, 738–746.
16. Figueiredo, P.A., Spera, K.D., Gomes, A. da C., Dokkedal, A.L., Saldanha, L.L., Ximenes, V.F., Silva, L.P., da Silva, R.M.G., 2016. Antioxidant activity and chemical characterization of extracts from the Genus Hymenaea. Res. J. Med. Plant 10, 330–339. https://doi.org/10.3923/rjmp.2016.330.339
17. Galvão, M.A.M., Arruda, A.O. de, Bezerra, I.C.F., Ferreira, M.R.A., Soares, L.A.L., 2018. Evaluation of the Folin-Ciocalteu Method and Quantification of Total Tannins in Stem Barks and Pods from Libidibia ferrea (Mart. ex Tul) L. P. Queiroz. Brazilian Arch. Biol. Technol. 61. https://doi.org/10.1590/1678-4324-2018170586
18. Hassan, S.K., El-Sammad, N.M., Mousa, A.M., Mohammed, M.H., Farrag, A. el R.H., Hashim, A.N.E., VictoriaWerner, UlrikeLindequist, Nawwar, M.A.E.-M., 2015. Hypoglycemic and antioxidant activities of Caesalpinia ferrea Martius leaf extract in streptozotocin-induced diabetic rats 5.
19. Holanda, B.F., Araujo, D.F. de, Silva, J.N.R. da, Pereira, M.G., Pires, A. de F., Assreuy, A.M., 2021. Polysaccaride-rich extract of Caesalpina ferrea stem barks attenuates mice acute inflammation induced by zymosan: Oxidative stress modulation. J. Ethnopharmacol. 267.
20. Jayaprakasam, B., Alexander-Lindo, R.L., DeWitt, D.L., Nair, M.G., 2007. Terpenoids from Stinking toe (Hymneae courbaril) fruits with cyclooxygenase and lipid peroxidation inhibitory activities. Food Chem. 105, 485–490. https://doi.org/10.1016/j.foodchem.2007.04.004
21. Jenkins, T., Bhattacharyya, J., Majetich, G., Teng, Q., De Fatima, A.M., Almeida, R., 1999. Flavonoids from the root-bark of Dioclea grandiflora. Phytochemistry 52, 723–730. https://doi.org/10.1016/S0031-9422(99)00314-3
22. Kobayashi, Y.T. da S., Almeida, V.T. de, Bandeira, T., Alcântara, B.N. de, Silva, A.S.B. da, Barbosa, W.L.R., Silva, P.B. da, Monteiro, M.V.B., 2015. Phytochemical evaluation and wound healing potential of the fruit extract ethanolic of jucá (Libidibia ferrea) in wistar rats. Brazilian J. Vet. Res. Anim. Sci. 52.
23. Lemos, V.S., Dos Santos, M.H., Rabelo, L.A., Côrtes, S.F., 2002. Spectral assignments and reference data: Total assignments of 1H and 13C NMR spectra of a new prenylated flavanone from Dioclea grandiflora. Magn. Reson. Chem. 40, 793–794. https://doi.org/10.1002/mrc.1096
24. Luna, M.S.M., Paula, R.A. d., Costa, R.M.P.B., Silva, J.V. do. A.V. d., M.T.S.Correiaa, 2020. Bioprospection of Libidibia ferrea var. ferrea: Phytochemical properties and antibacterial activity. South African J. Bot. 130, 103–108. https://doi.org/10.1016/j.sajb.2019.12.013
25. Pedrosa, T. do N., Barros, A.O., Nogueira, J.R., Fruet, A.C., Rodrigues, I.C., Calcagno, D.Q., Smith, M. de A.C., de Souza, T.P., Barros, S.B. de M., de Vasconcellos, M.C., Silva, F.M.A. da, Koolen, H.H.F., Maria-Engler, S.S., Lima, E.S., 2016. Anti-wrinkle and anti-whitening effects of jucá (Libidibia ferrea Mart.) extracts. Arch. Dermatol. Res. 308, 643–654. https://doi.org/10.1007/s00403-016-1685-0
26. Pereira, D.L., Cunha, A.P.S. da, Fernandes, H.P., Patias, N.S., Sinhorin, A.P., Sinhorin, V.D.G., 2020. Avaliação antioxidante do extrato da semente de hymenaea courbaril l. (jatobá) em camundongos tratados com acetaminofeno. Rev. Cuba. Plantas Med. 25, 1–13.
27. Pickler, T.B., Lopes, K.P., Magalhães, S.A., Krueger, C.M.A., Martins, M.M., Filho, V.C., Jozala, A.F., Grotto, D., Gerenutti, M., 2019. Effect of Libidibia ferrea bark and seed in maternal reproductive and biochemical outcomes and fetal anomaly in rats. Birth Defects Res. 111, 863–871. https://doi.org/10.1002/bdr2.1520
28. Port’s, P. da S., Chiste, R.C., Godoy, H.T., Prado, M.A., 2013. The phenolic compounds and the antioxidant potential of infusion of herbs from the Brazilian Amazonian region. FOOD Res. Int. 53, 875–881. https://doi.org/10.1016/j.foodres.2013.02.010
29. Prazeres, L.D.K.T., Aragão, T.P., Brito, S.A., Almeida, C.L.F., Silva, A.D., Mirella M. F. de Paula, Farias, J.S., Vieira, L.D., Damasceno, B.P.G.L., Rolim, L.A., Veras, B.O., Rocha, I.G., Neto, J.C.S., Bittencourt, M.L.F., Gonçalves, R. de C.R., Kitagawa, R.R., Wanderley, A.G., 2019. Antioxidant and Antiulcerogenic Activity of the Dry Extract of Pods of Libidibia ferrea Mart. ex Tul. (Fabaceae). Oxid. Med. Cell. Longev. 2019, 1983137.
30. Silva, L.C.N. da, da Silva, C.A.J., de Souza, R.M., José Macedo, A., da Silva, M.V., dos Santos Correia, M.T., 2011. Comparative analysis of the antioxidant and DNA protection capacities of Anadenanthera colubrina, Libidibia ferrea and Pityrocarpa moniliformis fruits. Food Chem. Toxicol. 49, 2222–2228. https://doi.org/10.1016/j.fct.2011.06.019
31. Silva, R.W.V. da, Martins, G.M.G., Nascimento, R.A. do, Viana, A.F. da S., Aguiar, F.S. de, Silva, B.A. da, 2019. Uso da metodologia de superfície de resposta na otimização da extração de compostos fenólicos da casca dos frutos de Hymenaea courbaril L. (Jatobá). Brazilian J. Food Technol. 22, 1–13. https://doi.org/10.1590/1981-6723.08918
32. Spera, K.D., Figueiredo, P.A., Santos, P.C.E.E., Barbosa, F.C., Alves, C.P., Dokkedal, A.L., Saldanha, L.L., Silva, L.P., Figueiredo, C.R., Ferreira, P.C., Da Silva, R.M.G.G., 2019. Genotoxicity, anti-melanoma and antioxidant activities of hymenaea courbaril L. Seed extract. An. Acad. Bras. Cienc. 91. https://doi.org/10.1590/0001-3765201920180446

**Studies on Antioxidant Activity**

1. Andrade, B. de A., Corrêa, A.J.C., Gomes, A.K.S., Neri, P.M. da S., Sobrinho, T.J. da S.P., Araújo, T.A. de S., Castro, V.T.N. de A. e, Amorim, E.L.C. de, 2019. Photoprotective activity of medicinal plants from the caatinga used as anti-inflammatories. Pharmacogn. Mag. 15, 356–361.
2. Azevedo, L.F.C. de, Ferreira, T.A.A., Melo, K.M., Dias, C.L.P., Bastos, C.E.M.C., Santos, S.F., Alberdan da Silva Santos, Nagamachi, C.Y., Pieczarka, J.C., 2020. Aqueous ethanol extract of Libidibia ferrea (Mart. Ex Tul) L.P. Queiroz (juca) exhibits antioxidant and migration-inhibiting activity in human gastric adenocarcinoma (ACP02) cells. PLoS One 15, e0226979.
3. Barros, A.O., De Souza, R.S., Aranha, E.S.P., Da Costa, L.M., De Souza, T.P., De Vasconcellos, M.C., Lima, E.S., 2014. Antioxidant and hepatoprotective activities of libidibia ferrea bark and fruit extracts. Int. J. Pharm. Pharm. Sci. 6, 71–76.
4. Bezerra, G.P., Góis, R.W.D.S., Brito, T.S. De, Lima, F.J.B. De, Bandeira, M.A.M., Romero, N.R., Magalhães, P.J.C., Santiago, G.M.P., 2013. Phytochemical study guided by the myorelaxant activity of the crude extract, fractions and constituent from stem bark of Hymenaea courbaril L. J. Ethnopharmacol. 149, 62–69. https://doi.org/10.1016/j.jep.2013.05.052
5. Botelho, F. V., Alvarez-Leite, J.I., Lemos, V.S., Pimenta, A.M.C.C., Calado, H.D.R.R., Matencio, T., Miranda, C.T., Pereira-Maia, E.C., 2007. Physicochemical study of floranol, its copper(II) and iron(III) complexes, and their inhibitory effect on LDL oxidation. J. Inorg. Biochem. 101, 935–943. https://doi.org/10.1016/j.jinorgbio.2007.03.007
6. Falcão, T.R., Araújo, A.A. de, Soares, L.A.L., Farias, I.B. de, Silva, W.A.V. da, Ferreira, M.R.A., Jr, R.F. de A., Medeiros, J.S. de, Lopes, M.L.D. de S., Guerra, G.C.B., 2019a. Libidibia ferrea fruit crude extract and fractions show anti-inflammatory, antioxidant, and antinociceptive effect in vivo and increase cell viability in vitro. Evidence-based Complement. Altern. Med. https://doi.org/https://doi.org/10.1155/2019/6064805
7. Falcão, T.R., Rodrigues, C.A.O., De Araújo, A.A., de Medeiros, C.A.C.X., Soares, L.A.L., Ferreira, M.R.A., Vasconcelos, R.C., De Araújo Júnior, R.F., De Sousa Lopes, M.L.D., Guerra, G.C.B., Falcao, T.R., Oliveira Rodrigues, C.A., de Araujo, A.A., de Medeiros, C.A.C.X., Lira Soares, L.A., Assuncao Ferreira, M.R., Carvalho Vasconcelos, R., de Araujo Junior, R.F., De Sousa Lopes, M.L.D., Bernardo Guerra, G.C., 2019b. Crude extract from Libidibia ferrea (Mart. ex. Tul.) LP Queiroz leaves decreased intra articular inflammation induced by zymosan in rats. BMC Complement. Med. Ther. 19. https://doi.org/10.1186/s12906-019-2454-3
8. Farias, D.F., Souza, T.M., Viana, M.P., Soares, B.M., Cunha, A.P., Vasconcelos, I.M., Ricardo, N.M.P.S., Ferreira, P.M.P., Melo, V.M.M., Carvalho, A.F.U., 2013. Antibacterial, antioxidant, and anticholinesterase activities of plant seed extracts from Brazilian semiarid region. BioMed Res. Int. 2013.
9. Figueiredo, P.A., Spera, K.D., Gomes, A. da C., Dokkedal, A.L., Saldanha, L.L., Ximenes, V.F., Silva, L.P., da Silva, R.M.G., 2016. Antioxidant activity and chemical characterization of extracts from the Genus Hymenaea. Res. J. Med. Plant 10, 330–339. https://doi.org/10.3923/rjmp.2016.330.339
10. Hassan, S.K., El-Sammad, N.M., Mousa, A.M., Mohammed, M.H., Farrag, A. el R.H., Hashim, A.N.E., VictoriaWerner, UlrikeLindequist, Nawwar, M.A.E.-M., 2015. Hypoglycemic and antioxidant activities of Caesalpinia ferrea Martius leaf extract in streptozotocin-induced diabetic rats 5.
11. Holanda, B.F., Araujo, D.F. de, Silva, J.N.R. da, Pereira, M.G., Pires, A. de F., Assreuy, A.M., 2021. Polysaccaride-rich extract of Caesalpina ferrea stem barks attenuates mice acute inflammation induced by zymosan: Oxidative stress modulation. J. Ethnopharmacol. 267.
12. Jayaprakasam, B., Alexander-Lindo, R.L., DeWitt, D.L., Nair, M.G., 2007. Terpenoids from Stinking toe (Hymneae courbaril) fruits with cyclooxygenase and lipid peroxidation inhibitory activities. Food Chem. 105, 485–490. https://doi.org/10.1016/j.foodchem.2007.04.004
13. Pereira, D.L., Cunha, A.P.S. da, Fernandes, H.P., Patias, N.S., Sinhorin, A.P., Sinhorin, V.D.G., 2020. Avaliação antioxidante do extrato da semente de hymenaea courbaril l. (jatobá) em camundongos tratados com acetaminofeno. Rev. Cuba. Plantas Med. 25, 1–13.
14. Port’s, P. da S., Chiste, R.C., Godoy, H.T., Prado, M.A., 2013. The phenolic compounds and the antioxidant potential of infusion of herbs from the Brazilian Amazonian region. FOOD Res. Int. 53, 875–881. https://doi.org/10.1016/j.foodres.2013.02.010
15. Prazeres, L.D.K.T., Aragão, T.P., Brito, S.A., Almeida, C.L.F., Silva, A.D., Mirella M. F. de Paula, Farias, J.S., Vieira, L.D., Damasceno, B.P.G.L., Rolim, L.A., Veras, B.O., Rocha, I.G., Neto, J.C.S., Bittencourt, M.L.F., Gonçalves, R. de C.R., Kitagawa, R.R., Wanderley, A.G., 2019. Antioxidant and Antiulcerogenic Activity of the Dry Extract of Pods of Libidibia ferrea Mart. ex Tul. (Fabaceae). Oxid. Med. Cell. Longev. 2019, 1983137.
16. Silva, L.C.N. da, da Silva, C.A.J., de Souza, R.M., José Macedo, A., da Silva, M.V., dos Santos Correia, M.T., 2011. Comparative analysis of the antioxidant and DNA protection capacities of Anadenanthera colubrina, Libidibia ferrea and Pityrocarpa moniliformis fruits. Food Chem. Toxicol. 49, 2222–2228. https://doi.org/10.1016/j.fct.2011.06.019
17. Spera, K.D., Figueiredo, P.A., Santos, P.C.E.E., Barbosa, F.C., Alves, C.P., Dokkedal, A.L., Saldanha, L.L., Silva, L.P., Figueiredo, C.R., Ferreira, P.C., Da Silva, R.M.G.G., 2019. Genotoxicity, anti-melanoma and antioxidant activities of hymenaea courbaril L. Seed extract. An. Acad. Bras. Cienc. 91. https://doi.org/10.1590/0001-3765201920180446
