# Supplementary material for: Culture matters: A systematic review of antioxidant potential of tree legumes in the semiarid region of Brazil and local processing techniques as a driver of bioaccessibility
Source: PLoS One. 2022 Mar 9;17(3):e0264950. doi: 10.1371/journal.pone.0264950 (PMC8906597; doi:10.1371/journal.pone.0264950)
Supplement: Attachment — Submitted filename: PLOS Response to Reviewers.docx [file pone.0264950.s003.docx]

Response to Reviewers

Dear Editor,

We are very thankful to the opportunity of improving our paper and would like to thank the reviewers’ effort in reviewing our manuscript. We have considered comments and present a revised form of the paper. We answered to all the reviewers’ comments point by point, as following:

ACADEMIC EDITOR:

Thank you for submitting your revised manuscript to PLOS ONE. We believe it has merit but does not entirely fit PLOS ONE's publication standards as they currently stand and as suggested by Reviewer #1. As such, we invite you to submit an updated version of the manuscript as soon as possible that addresses the points mentioned by Reviewer #1 in order for us to make a decision on your article. Additionally, we request that you make appropriate changes to the manuscript's formatting and reference section to ensure compliance with the journal's guidelines (please also refer to the Instructions for authors)

*Answer: We have made the appropriate changes to the manuscript's formatting and reference section according with the journal's guidelines*

Journal Requirements:

When submitting your revision, we need you to address these additional requirements.

1. Please ensure that your manuscript meets PLOS ONE's style requirements, including those for file naming. The PLOS ONE style templates can be found at

<https://journals.plos.org/plosone/s/file?id=wjVg/PLOSOne_formatting_sample_main_body.pdf> and <https://journals.plos.org/plosone/s/file?id=ba62/PLOSOne_formatting_sample_title_authors_affiliations.pdf>

Answer: It was checked, and adjustments were done.

2. In your Data Availability statement, you have not specified where the minimal data set underlying the results described in your manuscript can be found. PLOS defines a study's minimal data set as the underlying data used to reach the conclusions drawn in the manuscript and any additional data required to replicate the reported study findings in their entirety. All PLOS journals require that the minimal data set be made fully available. For more information about our data policy, please see <http://journals.plos.org/plosone/s/data-availability>.

Answer: All relevant data are within the manuscript and its supporting information files.

3. Thank you for stating the following in the Acknowledgments Section of your manuscript:

“We thank teacher Jonathan who reviewed our English and polished our writing. We also thank the National Institutes of Science and Technology in Ethnobiology, Bioprospecting, and Nature Conservation, certified by CNPq, with financial support from Facepe, the Foundation for Support to Science and Technology of the State of Pernambuco (Grant number: APQ-0562-2.01/17). This funding source had no role in the design of this study, nor in its execution, analyses, and interpretation of the data, and no role in our decision to submit results.”

We note that you have provided additional information within the Acknowledgements Section that is not currently declared in your Funding Statement. Please note that funding information should not appear in the Acknowledgments section or other areas of your manuscript. We will only publish funding information present in the Funding Statement section of the online submission form.

Answer: We removed the additional information from the Acknowledgements Section.

Please remove any funding-related text from the manuscript and let us know how you would like to update your Funding Statement. Currently, your Funding Statement reads as follows:

“The National Institutes of Science and Technology in Ethnobiology, Bioprospecting, and Nature Conservation, certified by CNPq, provided financial support from Facepe, the Foundation for Support to Science and Technology of the State of Pernambuco to UPA (Grant number: APQ-0562-2.01/17). The funders had no role in study design, data collection and analysis, decision to publish, or preparation of the manuscript.”

Answer: Yes, we would like to update the Funding Statement as follows:

“The National Institutes of Science and Technology in Ethnobiology, Bioprospecting, and Nature Conservation, certified by CNPq, provided financial support from Facepe, the Foundation for Support to Science and Technology of the State of Pernambuco to UPA (Grant number: APQ-0562-2.01/17). The Brazilian Coordenação de Aperfeiçoamento de Pessoal de Nível financed the fee to publish this article (Finance Code 001). The funders had no role in study design, data collection and analysis, decision to publish, or preparation of the manuscript.”

4. Please review your reference list to ensure that it is complete and correct. If you have cited papers that have been retracted, please include the rationale for doing so in the manuscript text, or remove these references and replace them with relevant current references. Any changes to the reference list should be mentioned in the rebuttal letter that accompanies your revised manuscript. If you need to cite a retracted article, indicate the article’s retracted status in the References list and also include a citation and full reference for the retraction notice.

Answer: Yes, we checked.

Reviewer #1: I have carefully read the Ms entitled {Culture Matters: A Systematic Review of Antioxidant Potential of Tree Legumes in the Semiarid Region of Brazil and Local Processing Techniques as a Driver of Bio-accessibility}, the Ms is very interesting, well and enjoyable written, well designed, easy to follow and informative. The authors have done great efforts to extract such interesting data and recommendations especially the conditions changing the acceptability of phenolic compounds and its cultural relationship.

Answer: We are thankful for your comments and notes, they have contributed to improve our work. We hope to attend in a satisfactory way your recommendations.

I have some minor corrections

P2 correct “ (e.g( consisdine et al., 2017) ”

Answer: It was corrected.

P2 correct “ (Ferreira-Júnior, W; Campos, L; Pieroni, A; Albuquerque, 2018) ”

Answer: It was corrected.

P2L13, delete “see”

Answer: It was deleted.

P3 correct “ (Ghoshal, 2018))”

Answer: It was corrected.

Page 4 delete “see”

Answer: It was deleted.

P5l3 rephrase “we synthesize the known ……..etc”

Answer: It was rephrased.

“In short, in this study, by reviewing research from different areas, we summarize the current knowledge about the antioxidant potential of these plants and the likely effects of several processing techniques on this potential”

P6 no need to mention the names of the authors inside the experimental description (Data extraction section), it might be confusing to the respective reader; please rephrase this section

Answer: It was rephrased.

“With the assistance of the tool Rayyan, all records were organized and deleted duplicates were deleted. Using the same tool, initially, titles and abstracts underwent a first screening, at which point we excluded those that did not meet the selection criteria. Then, we proceeded to a full reading of potentially eligible texts.”

P8 L13-16, please add these data into table footnote

Answer: It was replaced into table footnote.

P49 correct (2013)found

Answer: It was corrected.

Carefully check the format of scientific names inside the reference section, it should be italic ( e.g

Answer: It was checked and corrected.

Please correct Fig 2 legend (Page 8) and add symbols on the photos (a-i) not to describe as legend read from top-down as it might be confusing for some reader

Answer: It was corrected.

Reviewer #2: Revised format of the paper Culture Matters: A Systematic Review of Antioxidant Potential of Tree Legumes in the Semiarid Region of Brazil and Local Processing Techniques as a Driver of Bioaccessibility is in an acceptable form as it meets the standards of the journal furthermore the language of the paper fulfils the requirement of the journal. The paper in its present format may be accepted for publication.

Answer: We are thankful for your comments.

After responding to all comments on our paper we are now resubmitted the new version of our manuscript to this journal and look forward to your positive response.

Best regards,

Juliana Maia

(on behalf of the authors)
